# The Shadow of Bias

**DOI:** 10.1371/journal.pbio.1001608

**Published:** 2013-07-16

**Authors:** Jonathan M. Chase

**Affiliations:** Freelance Science Writer, Saint Louis, Missouri, United States of America

Recent philosophical dissection of the scientific method can be caricatured as a polar debate between Karl Popper's sober view of objective development and falsification of hypotheses and Thomas Kuhn's more glamorous espousal of a role for ideology and subjectivity; “real science” as performed by the authors of this and other journals is probably a rich mix of the two.

But while scientific ideology is arguably a necessary ingredient for paradigm shifts (a phrase coined by Kuhn himself), it has an unfortunate flipside. Although cases of overt scientific fraud are thankfully quite rare and actively policed by scientists, administrators, and funding agencies, there are many more subtle ways by which scientific results are influenced by ideologies, leading to bias in what is reported in the literature. It is well known, for example, that results in support of a given hypothesis are more likely to be published in a “higher impact” journal than are negative results, leading to what's known as “publication bias.” Even within a study, bias can emerge from the choice of experimental design and/or the presentation and analysis of the results. Such bias is clearly counterproductive to scientific progress, but few scientists can reasonably claim to have never succumbed to at least some bias in their studies.

In biomedical science, animal models are essential to triage possible therapeutic interventions for human diseases prior to possible clinical trials. However, such studies might also be particularly prone to biases from scientists who have personal, professional, and financial incentives to publish important and exciting results. As a result, dozens of studies are often published that examine the effect of a particular intervention on animal models. Because experimental outcomes of the same treatment are often quite variable, meta-analysis can be used to combine all studies of that intervention into a single analysis. Meta-analysis takes the magnitude of difference between treatments (e.g., drug versus placebo), known as an effect size, from a single study, and then combines effect sizes between studies on the same topic (along with estimates of sample size and variance) to allow detection of the overall magnitude of effect. In this way, even if studies give somewhat conflicting answers to the same treatment, an overall effect among studies can be calculated to achieve a more conclusive answer.

While meta-analysis is a powerful tool to overcome the variation among studies and arrive at an answer to a particular scientific question (e.g., does a particular intervention alleviate the symptoms of a disease?), it is less powerful in its ability to detect publication bias and the selective presentation of analyses. In the biomedical sciences, such biases not only slow the progression of science, but they could also result in bringing ineffective or harmful substances to clinical trial, creating considerable financial and health costs. Thus, it is important to understand just how rampant these biases are.

In the current issue of PLOS Biology, Tsilidis and colleagues take the bold step of examining bias by employing a relatively new type of approach—a sort of meta-analysis of meta-analyses. This allowed them to assess whether the numbers of studies finding statistically significant effects of a biomedical intervention were higher than what would be expected if there were no bias. Specifically, they analyzed 160 separate meta-analyses comprising more than 1,000 studies that used animal models to evaluate the efficacy of interventions of six major neurological disorders—Alzheimer disease, multiple sclerosis, two types of stroke, Parkinson disease, and spinal cord injury—4,445 comparisons in all. A large proportion of these meta-analyses (nearly 70%) reported an overall positive effect of the tested interventions on the affliction. However, most of these meta-analyses also reported a very large amount of variation among studies, indicating uncertainty about the true effect size. In addition, nearly half of the meta-analyses were influenced by a “small study effect,” where studies with smaller sample sizes had substantially different effect sizes than those with larger sample sizes.

To take their analysis to the next level, Tsilidis and colleagues examined the amount of “excess significance,” which asks whether the observed number of studies in a meta-analysis that gave statistically significant results is greater than would be expected under a plausible scenario with no bias. To define plausibility, the authors took the study with the lowest standard error as being the most precise and thus closest to the true effect size. Over all 4,445 comparisons, the observed number of significant results (1,719) was nearly twice the expected number (919), indicating considerable bias. Such bias was present in studies on all six neurological disorders and when analyzing the data in a number of different ways, including relaxing the assumptions of the true effect size.

In all, Tsilidis and colleagues suggested that only 30% of the 160 meta-analyses they examined showed a significant response to an intervention but had no small sample effects or excess significance. And of those, only eight had a sample size of more than 500 animals, leading the authors to conclude that a large proportion of biomedical studies, at least on these six important neurological disorders, were strongly biased towards finding larger effects of interventions than truly exist. From this, they make the important observation that although inherent differences between animals and humans certainly plays a role, biases towards finding positive effects might explain a significant number of cases where seemingly promising interventions from animal studies failed in clinical trials with humans.

In the recipe for good science, a pinch of Kuhnian ideology allows paradigms to shift. However, the results of Tsilidis and colleagues emphasize that this pendulum can swing too far and that a healthy dose of Popperian falsifiability is necessary to restrict the inevitable creep of bias into scientific endeavor. With increasing numbers of humans afflicted with neurological disorders, millions of animals sacrificed in the name of research, and billions of dollars spent on health care, it is imperative that biomedical scientists take action to alleviate these biases. Tsilidis and colleagues advocate a number of such actions, including the development of standard reporting protocols, preregistration of experimental design, and provisioning of raw data to the broader community, all of which should allow more efficient development of disease interventions from animal models to clinical trials.

**Tsilidis KK, Panagiotou OA, Sena ES, Aretouli E, Evangelou E, et al. (2013) Evaluation of Excess Significance Bias in Animal Studies of Neurological Diseases. doi:10.1371/journal.pbio.1001609**

